# First record of the spider genus *Hellamalthonica* Bosmans, 2023 (Araneae, Agelenidae) from Turkiye, with the description of a new species

**DOI:** 10.3897/BDJ.13.e157512

**Published:** 2025-07-16

**Authors:** Gökhan Gündüz, Rahşen S. Kaya

**Affiliations:** 1 Bursa Uludağ University Graduate School of Natural and Applied Sciences, Zoology Section, Bursa, Turkiye Bursa Uludağ University Graduate School of Natural and Applied Sciences, Zoology Section Bursa Turkiye; 2 Bursa Uludağ University, Faculty of Arts and Science, Department of Biology, TR-16059, Bursa, Turkiye Bursa Uludağ University, Faculty of Arts and Science, Department of Biology, TR-16059 Bursa Turkiye

**Keywords:** Ageleninae, Anatolia, Aranei, funnel-web, Marmara Region

## Abstract

**Background:**

The spider genus *Hellamalthonica* Bosmans, 2023 of the family Agelenidae currently contains five species and all species are known from Greece. This genus has not been recorded in Turkiye yet.

**New information:**

A new species, *Hellamalthonicakazdagensis* sp. nov., is described (♂♀) from Mount Kaz Dağı, Balıkesir Province, Turkiye. This is also the first report of *Hellamalthonica* in Turkiye. Morphological descriptions, photomicroscopy and Scanning Electron Microscopy (SEM) images of the new species, a key to all species of the genus and a distribution map of *Hellamalthonica* species are given.

## Introduction

The spider family Agelenidae C. L. Koch, 1837 currently includes 97 genera and 1420 extant species worldwide (WSC 2025). In Turkiye, 76 agelenid species belonging to 16 genera are known (Brignoli 1972, Brignoli 1978a, Brignoli 1978b, Demircan Aksan and Topçu 2022, Dimitrov et al. 2022, Zamani et al. 2024, WSC 2025). Although Turkiye represents a high diversity of agelenid fauna within the western Palaearctic Region, many taxa still remain unknown, including *Hellamalthonica* Bosmans, 2023. In recent years, the discovery of a new genus and several new species from Turkiye further highlights the significant agelenid diversity of the region (Kaya et al. 2023a, Kaya et al. 2023b, Zamani et al. 2024).

*Hellamalthonica* is a relatively small genus, currently known only from Greece. It was recently established by Bosmans (2023) for three species, previously placed within *Malthonica* Simon 1898, along with two newly-described species. Bosmans (2023) further noted that *Hellamalthonica* is closely related to the genus *Tegenaria* Latreille, 1804 differing from it only in the structures of the male palp and female epigyne.

Although there have been many recent taxonomic studies focusing on Agelenid spiders of Turkiye, many taxa still remain unknown. During the examination of spider material collected from Mount Kaz Dağı, we identified a new species belonging to *Hellamalthonica* which is described here. Additionally, a key to all known species of the genus is provided.

## Materials and methods

The present study is based upon the examination of one female and 11 male specimens belonging to the new species. The specimens were collected from the Mount Kaz Dağı, Balıkesir Province, located in the Marmara Region of Turkiye by pitfall traps and are kept in 70% ethanol. All samples were examined and measured with Leica S8 APO Stereomicroscope. Photographs were taken by a Leica MC170 HD camera attached to a Leica S8 APO stereomicroscope at the Arachnological Laboratory of Bursa Uludağ University. Measurements were taken with a micrometric ocular from the dorsal side of the palps and legs and are given in millimetres (mm). Lengths of leg segments are listed as: total length (femur, patella, tibia, metatarsus, tarsus). The epigyne was cleared in 10% potassium hydroxide (KOH) aqueous solution before photography. The left male palp was used for description and illustration. The colouration pattern is described, based on specimens preserved in 70% ethanol. The morphological terminology and leg spination formula follow Bolzern et al. (2013), Bolzern et al. (2009), Bolzern et al. (2008) and Bosmans (2023). The taxonomy and distribution follow WSC (2025).

For Scanning Electron Microscope (SEM) photography, the samples were first cleaned and dried for 15 minutes. Each sample was then attached to an aluminium stub using a sticky carbon disc. Then the samples were sputter-coated with a thin layer of gold-palladium for 2 minutes using a BAL-TEC SCD 005 sputter coater. The coated samples were placed in the SEM chamber for examination. Micrographs were captured with a ZEISS EVO 40 SEM, with a 20 kV acceleration voltage, at the SEM Laboratory of Bursa Uludağ University.

**Comparative examined material**

*Hellamalthonicaparaschiae* (Brignoli, 1984): female (holotype, Museo Civico di Storia Naturale of Verona Italy): Greece, Naxos, 04.05.1982, L. Paraschi leg. – examined.

The following abbreviations are used in the text and figures:

- Eyes: AER ‒ anterior eye row, ALE ‒ anterior lateral eye, AME ‒ anterior median eye, PER ‒ posterior eye row, PLE ‒ posterior lateral eye, PME ‒ posterior median eye.
- Spination: d ‒ dorsal, Fe ‒ femur, Mt ‒ metatarsus, Pa ‒ patella, pl ‒ prolateral, rl ‒ retrolateral, Ti ‒ tibia, v ‒ ventral.
- Male palp: C – conductor, E – embolus, Eb – base of the embolus, Rd – retrodorsal tibial apophysis, Rl – retrolateral tibial apophysis, Rv – retroventral tibial apophysis, Ta – tegular apophysis.
- Epigyne: Cd – copulatory duct, Co – copulatory opening, Et – epigynal teeth, Fd – fertilisation duct, Pr – primary receptacle, Sr – secondary receptacle.

**Depositories**

- **ZMUT** Zoological Museum of the University of Turku, Finland (V. Vahtera).
- **ZMUU** Zoological Museum of the Bursa Uludağ University, Turkiye (R.S. Kaya).

## Taxon treatments

### Hellamalthonica kazdagensis

Gündüz & Kaya sp. nov.

C23EA08A-9B7E-5E3A-A575-DC4CCCBCC0FC

85C8335C-B146-4E2C-8BEC-BCAC2A2D77A6

#### Materials

- **Type status:** Holotype. **Occurrence:** catalogNumber: G5; recordNumber: Hellkazd2025_1; individualCount: 1; sex: male; lifeStage: adult; preparations: 70% Ethanol Solution; occurrenceID: 0D75A6E9-6420-54B0-B152-410F75DBFB5C; **Taxon:** scientificName: *Hellamalthonicakazdagensis*; **Location:** country: Turkiye; stateProvince: Balıkesir; locality: Ayazma District, Mount Kaz Dağı; verbatimElevation: 491 m; verbatimLatitude: 39°44'46"N; verbatimLongitude: 26°50'27"E; georeferenceProtocol: GPS; **Identification:** identifiedBy: Gökhan Gündüz, Rahşen S. Kaya; **Event:** samplingProtocol: Pitfall Trap; eventDate: 07.10.2016-25.07.2017; **Record Level:** language: en; institutionID: ZMUU
- **Type status:** Paratype. **Occurrence:** catalogNumber: G6; recordNumber: Hellkazd2025_2; individualCount: 1; sex: female; lifeStage: adult; preparations: 70% Ethanol Solution; occurrenceID: A3854B12-DED5-5AB2-AAC0-20F6C8EDA506; **Taxon:** scientificName: *Hellamalthonicakazdagensis*; **Location:** country: Turkiye; stateProvince: Balıkesir; locality: Ayazma District, Mount Kaz Dağı; verbatimElevation: 491 m; verbatimLatitude: 39°44'46"N; verbatimLongitude: 26°50'27"E; georeferenceProtocol: GPS; **Identification:** identifiedBy: Gökhan Gündüz, Rahşen S. Kaya; **Event:** samplingProtocol: Pitfall Trap; eventDate: 07.10.2016-25.07.2017; **Record Level:** language: en; institutionID: ZMUU
- **Type status:** Paratype. **Occurrence:** catalogNumber: G7; recordNumber: Hellkazd2025_3; individualCount: 1; sex: male; lifeStage: adult; preparations: 70% Ethanol Solution; occurrenceID: EA2171EF-FE8D-5F4A-9D96-BFB4415B9691; **Taxon:** scientificName: *Hellamalthonicakazdagensis*; **Location:** country: Turkiye; stateProvince: Balıkesir; locality: Ayazma District, Mount Kaz Dağı; verbatimElevation: 491 m; verbatimLatitude: 39°44'46"N; verbatimLongitude: 26°50'27"E; georeferenceProtocol: GPS; **Identification:** identifiedBy: Gökhan Gündüz, Rahşen S. Kaya; **Event:** samplingProtocol: Pitfall Trap; eventDate: 07.10.2016-25.07.2017; **Record Level:** language: en; institutionID: ZMUU
- **Type status:** Paratype. **Occurrence:** catalogNumber: G8; recordNumber: Hellkazd2025_4; individualCount: 1; sex: male; lifeStage: adult; preparations: 70% Ethanol Solution; occurrenceID: B167FA78-7B98-5265-A87A-D4A945BBEA97; **Taxon:** scientificName: *Hellamalthonicakazdagensis*; **Location:** country: Turkiye; stateProvince: Balıkesir; locality: Ayazma District, Mount Kaz Dağı; verbatimElevation: 491 m; verbatimLatitude: 39°44'46"N; verbatimLongitude: 26°50'27"E; georeferenceProtocol: GPS; **Identification:** identifiedBy: Gökhan Gündüz, Rahşen S. Kaya; **Event:** samplingProtocol: Pitfall Trap; eventDate: 07.10.2016-25.07.2017; **Record Level:** language: en; institutionID: ZMUU
- **Type status:** Paratype. **Occurrence:** catalogNumber: G9; recordNumber: Hellkazd2025_5; individualCount: 1; sex: male; lifeStage: adult; preparations: 70% Ethanol Solution; occurrenceID: C7418D59-2C34-55B0-931B-CAC6C347A726; **Taxon:** scientificName: *Hellamalthonicakazdagensis*; **Location:** country: Turkiye; stateProvince: Balıkesir; locality: Ayazma District, Mount Kaz Dağı; verbatimElevation: 491 m; verbatimLatitude: 39°44'46"N; verbatimLongitude: 26°50'27"E; georeferenceProtocol: GPS; **Identification:** identifiedBy: Gökhan Gündüz, Rahşen S. Kaya; **Event:** samplingProtocol: Pitfall Trap; eventDate: 07.10.2016-25.07.2017; **Record Level:** language: en; institutionID: ZMUU
- **Type status:** Paratype. **Occurrence:** catalogNumber: G10; recordNumber: Hellkazd2025_6; individualCount: 1; sex: male; lifeStage: adult; preparations: 70% Ethanol Solution; occurrenceID: 581989B1-CEE6-5878-836F-BA4BC9BB9E55; **Taxon:** scientificName: *Hellamalthonicakazdagensis*; **Location:** country: Turkiye; stateProvince: Balıkesir; locality: Ayazma District, Mount Kaz Dağı; verbatimElevation: 491 m; verbatimLatitude: 39°44'46"N; verbatimLongitude: 26°50'27"E; georeferenceProtocol: GPS; **Identification:** identifiedBy: Gökhan Gündüz, Rahşen S. Kaya; **Event:** samplingProtocol: Pitfall Trap; eventDate: 07.10.2016-25.07.2017; **Record Level:** language: en; institutionID: ZMUU
- **Type status:** Paratype. **Occurrence:** catalogNumber: G11; recordNumber: Hellkazd2025_7; individualCount: 1; sex: male; lifeStage: adult; preparations: 70% Ethanol Solution; occurrenceID: AF465643-1635-5B51-910F-4BE4FC170515; **Taxon:** scientificName: *Hellamalthonicakazdagensis*; **Location:** country: Turkiye; stateProvince: Balıkesir; locality: Ayazma District, Mount Kaz Dağı; verbatimElevation: 491 m; verbatimLatitude: 39°44'46"N; verbatimLongitude: 26°50'27"E; georeferenceProtocol: GPS; **Identification:** identifiedBy: Gökhan Gündüz, Rahşen S. Kaya; **Event:** samplingProtocol: Pitfall Trap; eventDate: 07.10.2016-25.07.2017; **Record Level:** language: en; institutionID: ZMUU
- **Type status:** Paratype. **Occurrence:** catalogNumber: G12; recordNumber: Hellkazd2025_8; individualCount: 1; sex: male; lifeStage: adult; preparations: 70% Ethanol Solution; occurrenceID: 1F9B3C7A-8366-56A4-9FD6-71B713CD8BF3; **Taxon:** scientificName: *Hellamalthonicakazdagensis*; **Location:** country: Turkiye; stateProvince: Balıkesir; locality: Ayazma District, Mount Kaz Dağı; verbatimElevation: 491 m; verbatimLatitude: 39°44'46"N; verbatimLongitude: 26°50'27"E; georeferenceProtocol: GPS; **Identification:** identifiedBy: Gökhan Gündüz, Rahşen S. Kaya; **Event:** samplingProtocol: Pitfall Trap; eventDate: 07.10.2016-25.07.2017; **Record Level:** language: en; institutionID: ZMUU
- **Type status:** Paratype. **Occurrence:** catalogNumber: G13; recordNumber: Hellkazd2025_9; individualCount: 1; sex: male; lifeStage: adult; preparations: 70% Ethanol Solution; occurrenceID: B1E27E2A-52D0-5AB1-8F41-469C9399A470; **Taxon:** scientificName: *Hellamalthonicakazdagensis*; **Location:** country: Turkiye; stateProvince: Balıkesir; locality: Ayazma District, Mount Kaz Dağı; verbatimElevation: 491 m; verbatimLatitude: 39°44'46"N; verbatimLongitude: 26°50'27"E; georeferenceProtocol: GPS; **Identification:** identifiedBy: Gökhan Gündüz, Rahşen S. Kaya; **Event:** samplingProtocol: Pitfall Trap; eventDate: 07.10.2016-25.07.2017; **Record Level:** language: en; institutionID: ZMUU
- **Type status:** Paratype. **Occurrence:** catalogNumber: G14; recordNumber: Hellkazd2025_10; individualCount: 1; sex: male; lifeStage: adult; preparations: 70% Ethanol Solution; occurrenceID: 989F235F-93F1-50BF-B979-6EEE1E216278; **Taxon:** scientificName: *Hellamalthonicakazdagensis*; **Location:** country: Turkiye; stateProvince: Balıkesir; locality: Ayazma District, Mount Kaz Dağı; verbatimElevation: 491 m; verbatimLatitude: 39°44'46"N; verbatimLongitude: 26°50'27"E; georeferenceProtocol: GPS; **Identification:** identifiedBy: Gökhan Gündüz, Rahşen S. Kaya; **Event:** samplingProtocol: Pitfall Trap; eventDate: 07.10.2016-25.07.2017; **Record Level:** language: en; institutionID: ZMUU
- **Type status:** Paratype. **Occurrence:** catalogNumber: G15; recordNumber: Hellkazd2025_11; individualCount: 1; sex: male; lifeStage: adult; preparations: 70% Ethanol Solution; occurrenceID: B25369AA-4BF6-506C-9133-0931A4FF9EC0; **Taxon:** scientificName: *Hellamalthonicakazdagensis*; **Location:** country: Turkiye; stateProvince: Balıkesir; locality: Ayazma District, Mount Kaz Dağı; verbatimElevation: 491 m; verbatimLatitude: 39°44'46"N; verbatimLongitude: 26°50'27"E; georeferenceProtocol: GPS; **Identification:** identifiedBy: Gökhan Gündüz, Rahşen S. Kaya; **Event:** samplingProtocol: Pitfall Trap; eventDate: 07.10.2016-25.07.2017; **Record Level:** language: en; institutionID: ZMUT

#### Description

**Figure 1.**
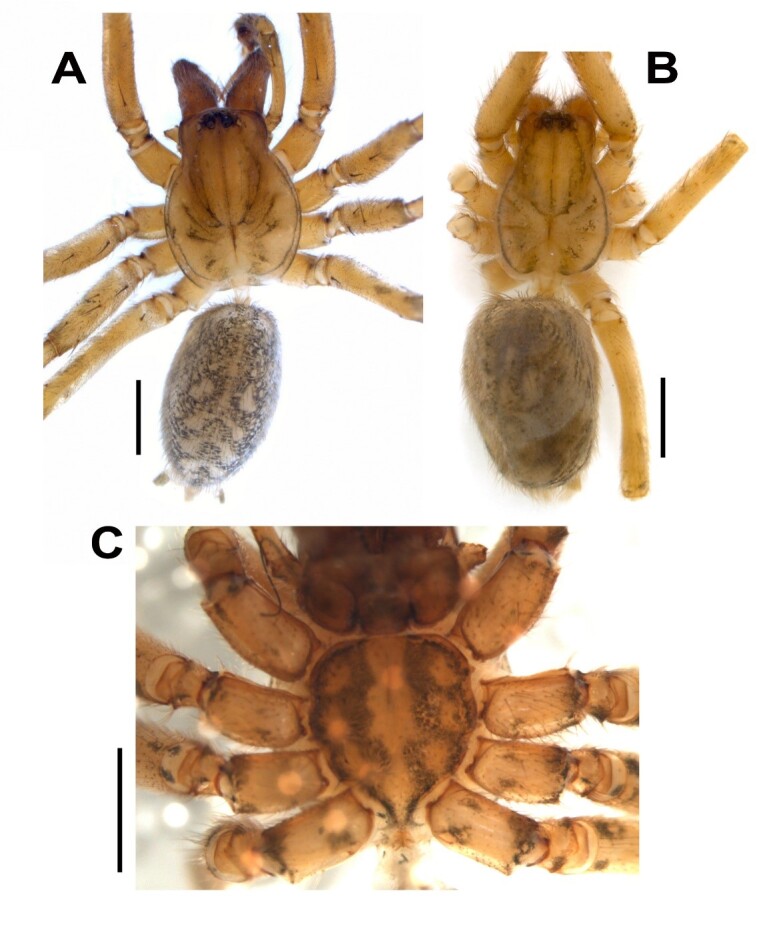
*Hellamalthonicakazdagensis* sp. nov. **A** Male habitus, dorsal; **B** Female habitus, dorsal; **C** Male, sternum and trochanters. Scale bars: 1 mm.

**Male (holotype, ZMUU).** Habitus as in Fig. 1A. Total length 5.20. Carapace 2.50 long, 1.90 wide. Carapace light brown, with two indistinct longitudinal bands, lighter in posterior and with radial dark lines, margins narrowly darkened, cephalic region darker; Eye sizes: AME: 0.06, ALE: 0.12, PME: 0.11, PLE: 0.12. Distance of PME–PME: 0.11; PME–AME: 0.07. Eye rows: AER 0.50 wide, PER 0.65 wide. AER slightly recurved, PER straight in dorsal view. Chelicerae: 1.70 long; 0.50 wide. Chelicerae light brown, with 3 promarginal and 3-4 retromarginal teeth. Sternum 1.50 long, 1.20 wide. Sternum, labium and maxillae brown. Sternum heart-shaped, pointed backwards, with a light median band and three pairs of lateral dots (Fig. 1C). Legs yellowish-brown, with annulations. Number of dorsal tarsal trichobothria on tarsi I- IV: 6 (in some of the male specimens the number of dorsal tarsal trichobothria on tarsi I- IV: 6-7). Measurements of legs: I: (3.0, 1.1, -, -, -), II: 8.3 (2.3, 0.9, 1.8, 2.2, 1.1), III: 7.3 (2.2, 0.8, 1.5, 1.9, 0.9), IV: 10.0 (2.6, 0.9, 2.4, 2.9, 1.2). Spination as given in Table 1. Abdomen 2.70 long; 1.50 wide. Abdomen dorsally greyish with yellowish-grey pattern, light greyish ventrally with dark spots. Spinnerets light greyish, darker basally.

**Figure 2.**
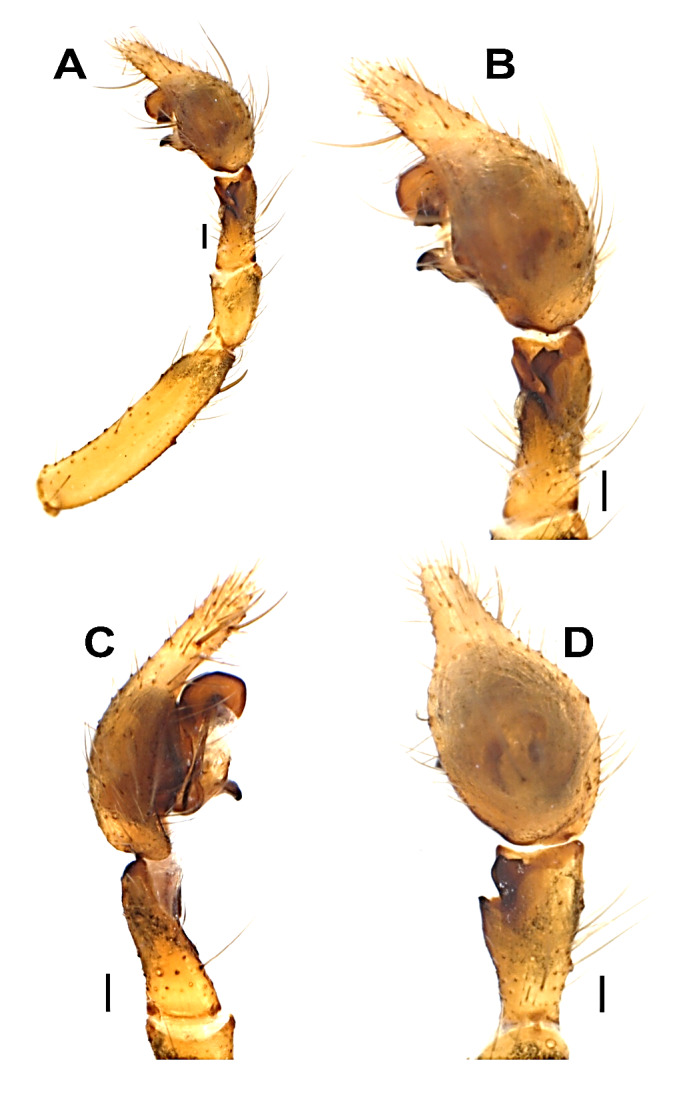
Male palp of *Hellamalthonicakazdagensis* sp. nov. **A–B** retrolateral view; **C** prolateral view; **D** dorsal view. Scale bars: 0.1 mm.

**Figure 3.**
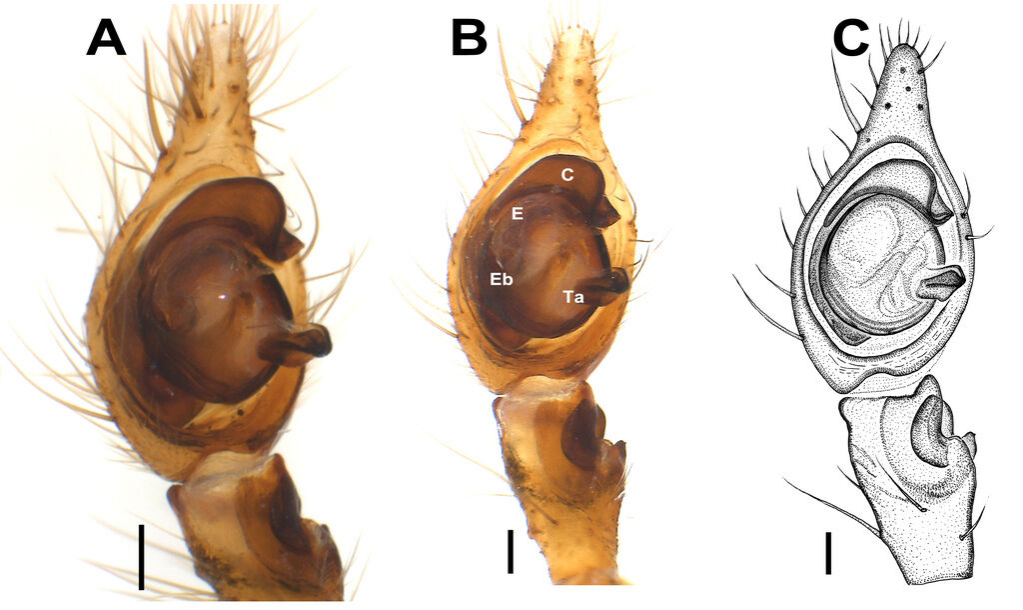
Male palp of *Hellamalthonicakazdagensis* sp. nov. **A–C** ventral view. Scale bars: 0.1 mm. Abbreviations: *C* – conductor, *E* – embolus, *Eb* – base of the embolus, *Ta* – tegular apophysis.

**Figure 4.**
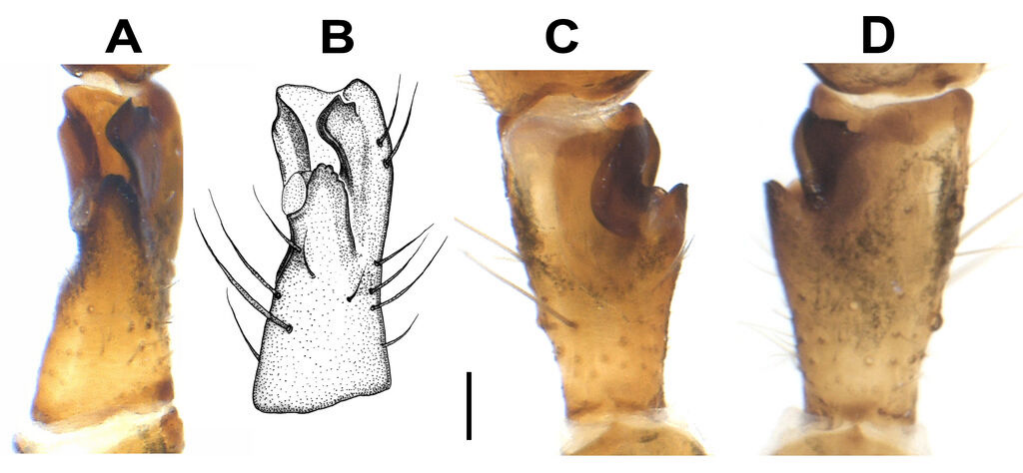
Male palpal tibia of *Hellamalthonicakazdagensis* sp. nov. **A–B** retrolateral view; **C** ventral view; **D** dorsal view. Scale bar: 0.1 mm.

**Figure 5.**
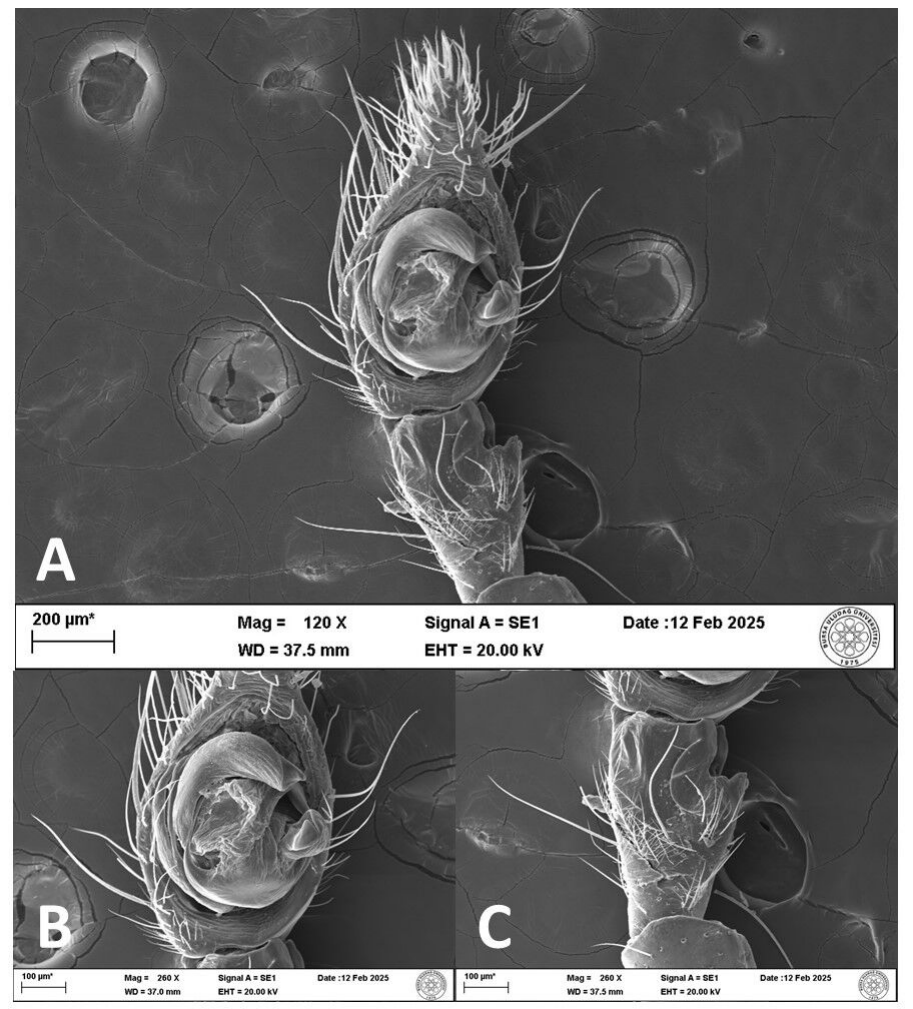
SEM micrographs of the male palp of *Hellamalthonicakazdagensis* sp. nov. **A–B** ventral view; **C** tibia, ventral view.

**Figure 6.**
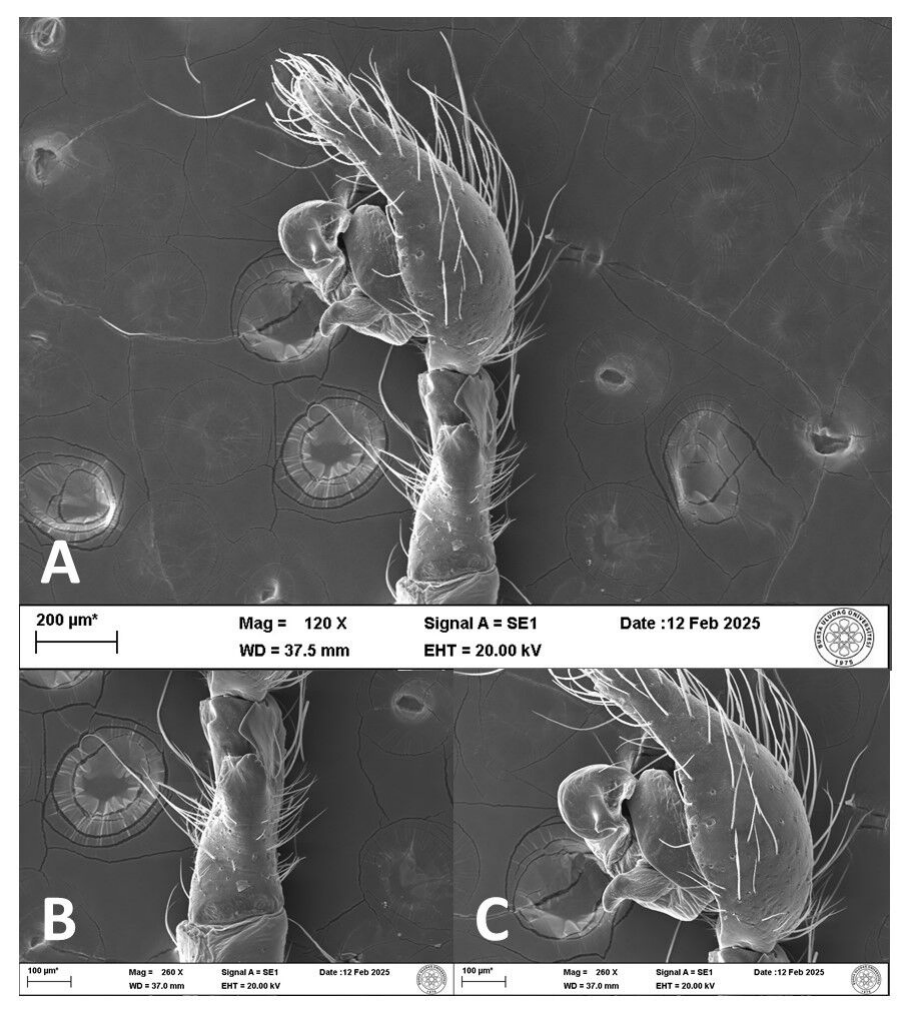
SEM micrographs of the male palp of *Hellamalthonicakazdagensis* sp. nov. **A, C** retrolateral view; **B** palpal tibia, retrolateral view.

**Figure 7.**
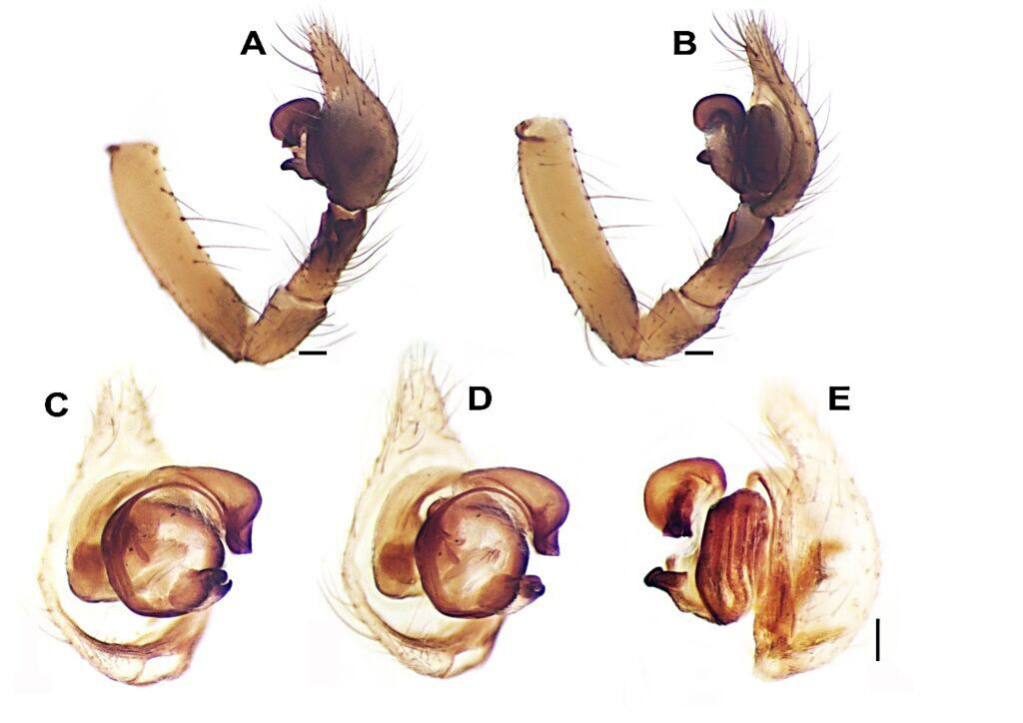
Paratype male palp and expanded bulb of *Hellamalthonicakazdagensis* sp. nov. **A–B** full palp, retrolateral view; **C–D** expanded bulbus, postero-ventral view; **E** expanded bulbus, retrolateral view. Scale bars: 0.1 mm.

**Palp** (Figs 2, 3, 4, 5, 6, 7). Femur (1.2 long) approximately 5.5 times longer than wide, approximately 2.2 times longer than tibia; patella 1.5 times longer than wide; tibia 2.5 times longer than wide, approximately 1.6 times shorter than cymbium; tibia with three apophyses: retrolateral (Rl), retroventral (Rv) and retrodorsal (Rd); retrolateral apophysis located at the mid-point of the tibia, rounded and tuberculated retrolaterally and triangular-shaped dorsally; retrodorsal apophysis located at the apical part of tibia, strongly sclerotised, more or less rectangular-shaped and distally V-truncated in dorsal view; retroventral apophysis as a ventral fold. Tibia with a distinct deep V-shaped prolateral notch. Cymbium as long as tibia +patella. Tegulum wide and broadly rounded. Tegular apophysis (Ta) originates at 3-4 o’clock position, plate-shaped and connected to the tegulum by a membranous structure; tip of the tegular apophysis partially split, with thickened edges. Conductor (C) curved C-shaped, roundly bent and transversal to cymbium; distal part conspicuously narrow and the proximal part gradually widens, rounding and folding, terminates at approximately ~ 2-3 o’clock position with simple and pointed or moderately truncated tip; the terminal end of the conductor is bifid, with the ventral branch forming a distinctively twisted structure, while the dorsal branch is separated by a thin, nearly transparent section and terminates in a sharply pointed tip; the base of the conductor not clearly detectable. Embolus (E) short, originates at 8-9 o’clock position with the tip at 1 o’clock position, thin basally, roundly bent and covered by the curling of conductor at approximately 11 o'clock position.

**Female**

Habitus as in Fig. 1B. Colouration as in male. Total length 4.30. Carapace 1.90 long, 1.30 wide. Eye sizes: AME: 0.05, ALE: 0.08, PME: 0.08, PLE: 0.08. Distance of PME–PME: 0.08; PME–AME: 0.07. Eye rows: AER 0.42 wide, PER 0.52 wide. AER slightly recurved, PER straight in dorsal view. Chelicerae 0.90 long, 0.35 wide. Sternum 1.20 long, 0.90 wide. Abdomen 2.40 long, 1.10 wide. Number of dorsal tarsal trichobothria on tarsi I: 6. Measurements of legs: I: 6.2 (1.6, 0.6, 1.6, 1.5, 0.9), II: (2.0, -, -, -, -), III: (1.5, -, -, -, -), IV: (2.1, -, -, -, -). Spination as given in Table 1.

**Figure 8.**
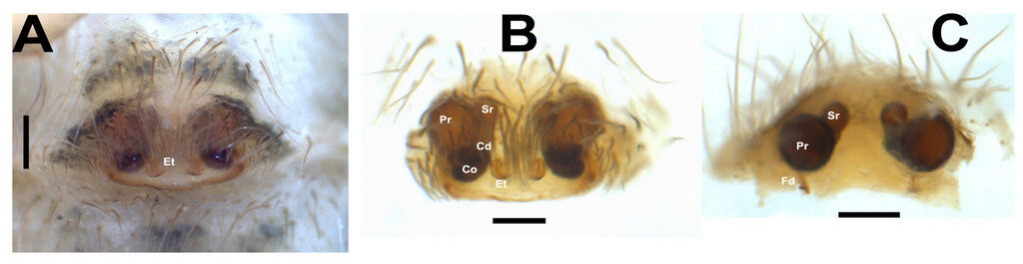
Epigyne of *Hellamalthonicakazdagensis* sp. nov. **A** intact epigyne, ventral view; **B** macerated epigyne, ventral view; **C** vulva, anterior view. Scale bars: 0.1 mm. Abbreviations: Cd – copulatory duct, Co – copulatory opening, Et – epigynal teeth, Fd – fertilisation duct , Pr – primary receptacle, Sr – secondary receptacle.

**Epigyne** (Fig. 8). Epigynal plate approximately 1.7 times wider than long, with straight and heavily sclerotised posterior margin, anterior part of the plate with a pair of concavities. Median plate with a pair of epigynal teeth (not well sclerotised), turning downwards; copulatory openings (Co) laterally positioned to epigynal teeth, copulatory ducts (Cd) wide, two pairs of receptacles (Pr and Sr) rounded, fertilisation duct (Fd) short and clearly visible.

#### Diagnosis

The male of the new species can be distinguished from all other *Hellamalthonica* species by the shape of the retrolateral tibial apophyses and the conductor. *Hellamalthonicakazdagensis* sp. nov. is closely related to *H.spinipalpis* and differs from the latter by the absence of short spines on tibia (vs. present) and square-shaped retrodorsal apophysis (Rd) (vs. spine-like). Females of the new species resemble those of *H.taigetos* in general appearance, but differ by the shape of the epigynal plate and the copulatory ducts (Cd). Additionally, the diameter of the secondary receptacles (Sr) is distinctly shorter: approximately one-third the diameter of the primary receptacle (Pr) in the new species, compared to about half in *H.taigetos*.

#### Etymology

The specific epithet “*kazdagensis*” refers to Mount Kaz Dağı, located in Balıkesir Province, north-western Turkiye, which constitutes the type locality of the species.

#### Distribution

Known only from the type locality in Balıkesir Province, north-western Turkiye (Figs 10, 11).

#### Ecology

The specimens were collected from Mount Kaz Dağı which is situated in the south-western part of the Marmara Region of Turkiye. The area has the characteristics of a transition zone between the typical Mediterranean and the Black Sea climates (Fig. 10).

#### Remarks

According to the diagnosis provided by Bosmans (2023), *Hellamalthonica* differs from both *Tegenaria* and *Malthonica* by the presence of a pair of tibial apophyses, the plate-like shape of the tegular apophysis (Ta) and the anterior position of the conductor (C) (see figs. 2A-O, 4A-B, 4E-F and 5A-B in Bosmans (2023)). In females, it is distinguished by the presence of a pair of epigynal teeth (Et) and two pairs of receptacles (see figs. 3A-H, 4C-D, 4G-H and 5C-D in Bosmans (2023)). However, based on the currently recognised *Tegenaria* species, the specimens examined in this study and the illustrations and descriptions provided by Bosmans (2023), we propose a revised diagnosis for the genus *Hellamalthonica*.

*Hellamalthonica* closely resembles *Tegenaria* in both somatic and copulatory organ characteristics. Shared features include relatively straight eye rows in both frontal and dorsal views, straight or slightly curved trochanters, patellae bearing only dorsal spines, tarsi without ventral spines, chelicerae with three promarginal and three to four subequal retromarginal teeth and a male palp with a complex retrolateral tibial apophysis (RTA) typically divided into two or three branches. However, it differs from *Tegenaria* by the following characteristics:

Somatic characters: One of the most distinctive somatic characteristics of the genus is the length of the chelicerae in males. Compared to *Tegenaria* species, the chelicerae of *Hellamalthonica* species, including the newly-described species *Hellamalthonicakazdagensis* sp. nov., are significantly longer in males (its length is approximately 3.4 times its width for *Hellamalthonicakazdagensis* sp. nov.).

Male palp: We propose that the number of retrolateral tibial apophyses should not be considered as a definitive taxonomic character, as this feature demonstrates variability (amongst *Tegenaria* species, which may possess either two or three such apophyses). Notably, *H.kazdagensis* sp. nov. is characterised by the presence of three retrolateral tibial apophyses, a trait that sets it apart from all currently recognised species within the genus *Hellamalthonica*. More significantly, the morphology of the retroventral apophysis (Rv) appears to offer greater taxonomic value. In both previously described *Hellamalthonica* species and *H.kazdagensis* sp. nov., the retroventral apophysis extends prolaterally as an almost two-dimensional fold, which may represent a key diagnostic feature. Furthermore, we concur with Bosmans (2023) in recognising the presence of short, stout spines on the retrolateral side as an important character for understanding interspecific relationships within *Hellamalthonica*. The tegular region in this genus is notably broad and distinctly oval. The tegular apophysis (Ta) is plate-like in form, typically bearing a notched tip. The conductor (C) is connected to the tegular region via a membranous section, broadens anteriorly and is displaying a transverse orientation. Embolus (E) is filiform and tapers gradually towards the tip.

**Figure 9.**
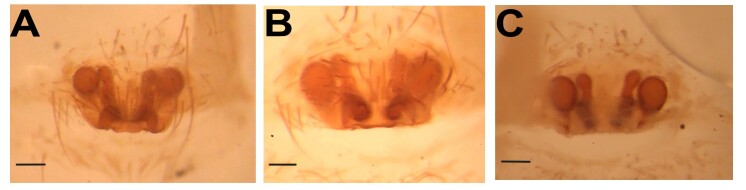
Epigyne of *Hellamalthonicaparaschiae* (Brignoli, 1984) (holotype). **A** macerated epigyne, ventral view; **B** macerated epigyne, ventral view; **C** vulva, dorsal view. Scale bars: 0.1 mm.

**Figure 10.**
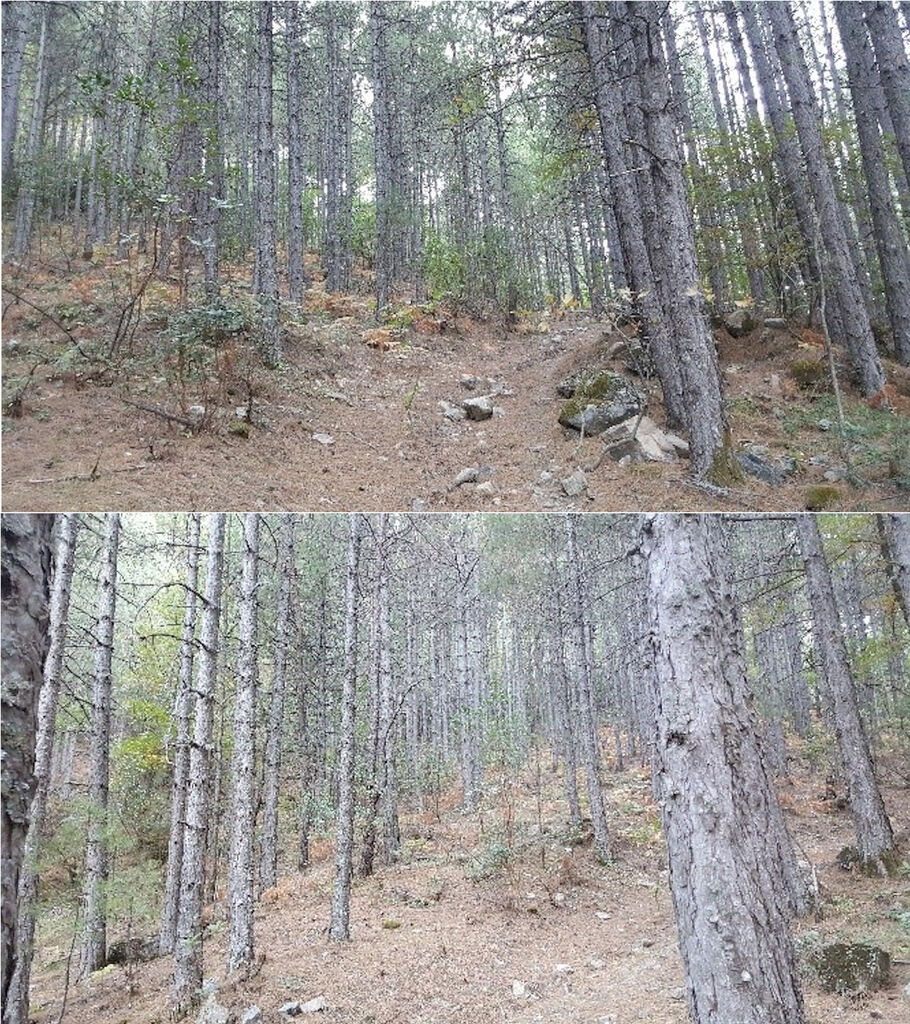
Habitats of *Hellamalthonicakazdagensis* sp. nov. in Mount Kaz Dağı, Balıkesir Province, Turkiye (Photo by E. A. Yağmur).

**Figure 11.**
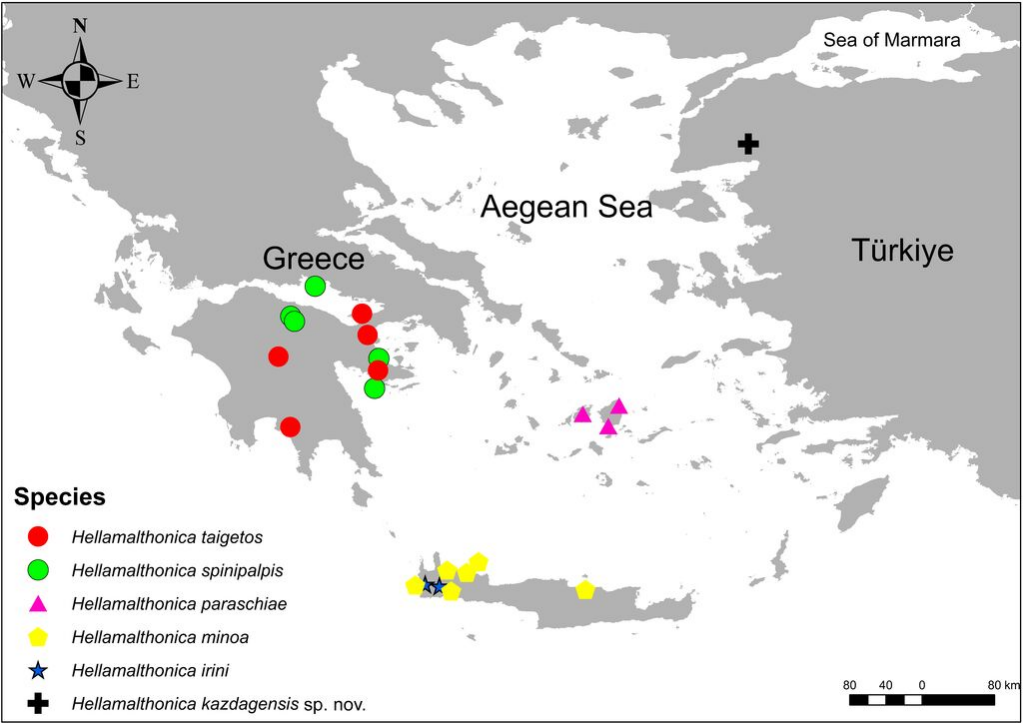
Distribution map of *Hellamalthonica* species.

Epigyne: In females of *Hellamalthonica*, the epigynal plate is roughly anchor-shaped with the exception of *H.paraschiae* (*Fig. 9*). A pair of epigynal teeth (Et) is located approximately at the middle of the plate. The copulatory openings (Co) are located at the lateral edges of the posterior margin and slightly directed upwards. The copulatory ducts (Cd) are curved and relatively broad. Before reaching the primary receptacle (Pr), the ducts first lead to a smaller, secondary receptacle (Sr). This secondary receptacle opens into the primary receptacle via a short transitional region.

## Identification Keys

### Key to males of *Hellamalthonica* species

**Table:** 

1	Palpal tibia with 3 tibial apophyses	*Hellamalthonicakazdagensis* sp. nov.
–	Palpal tibia with 2 tibial apophyses	2
2	Palpal tibia with stout denticles	3
–	Palpal tibia without denticles	4
3	Palpal retrodorsal tibial apophysis strongly curved; base wide, basal part approximately 2 times wider than the middle part	*Hellamalthonicaspinipalpis*
–	Palpal retrodorsal tibial apophysis slightly curved; base not wide, basal part approximately as wide as the middle part	*Hellamalthonicataigetos*
4	Conductor large and strongly curved; retrodorsal tibial apophysis small, pointed and with a small notch on the dorsal edge	*Hellamalthonicaparaschiae*
–	Conductor smaller and slightly curved; retrodorsal tibial apophysis larger	5
5	Tip of conductor extending retrolaterally; retrodorsal tibial apophysis long, thin and sharply pointed	*Hellamalthonicairini*
–	Tip of conductor extending apically; retrodorsal tibial apophysis short, thick and blunt	*Hellamalthonicaminoa*

### Key to females of *Hellamalthonica* species

**Table:** 

1	Epigynal plate anchor-shaped; copulatory openings located laterally	2
–	Epigynal plate not anchor-shaped; copulatory openings located medially	*Hellamalthonicaparaschiae*
2	Median plate wide in centrally (approximately as wide as the posterior edge of the median plate)	3
–	Median plate narrow in centrally (approximately half the width or less than the posterior edge of the median plate)	4
3	Epigynal plate approximately twice as wide as long	*Hellamalthonicaminoa*
–	Epigynal plate approximately as wide as long	*Hellamalthonicairini*
4	Secondary receptacles positioned at the same level as the primary receptacles	5
–	Secondary receptacles positioned more posteriorly than the primary receptacles	*Hellamalthonicaspinipalpis*
5	Diameter of the secondary receptacle approximately half the diameter of the primary receptacle	*Hellamalthonicataigetos*
–	Diameter of the secondary receptacle approximately one-third of the diameter of primary receptacle	*Hellamalthonicakazdagensis* sp. nov.

## Discussion

*Hellamalthonica* was separated from *Malthonica* primarily based on differences in genital structures and certain somatic characteristics (Bosmans 2023). The genus currently includes *H.minoa*, *H.paraschiae*, *H.spinipalpis*, as well as the recently described *H.taigetos* and *H.irini* (Bosmans 2023, WSC 2025). Our observations indicate that the new species described here shares several characteristics with these species, particularly in the structure of the palp and epigyne. These similarities raise important questions regarding the evolutionary relationships between *Tegenaria* and *Hellamalthonica*, suggesting that further phylogenetic analyses are needed to clarify the boundaries between these genera.

Bosmans (2023) emphasised that *Hellamalthonica* is primarily distributed in Greece and nearby regions, highlighting its biogeographical importance. The presence of our newly-described species outside this core distribution area could indicate either an extension of the genus range or reflect cryptic diversity within the *Tegenariini*. Given the complex taxonomic history of these genera, it is crucial to integrate morphological data with molecular analyses to better resolve their phylogenetic relationships.

Recent taxonomic studies on Agelenidae in Turkiye have emphasised the high levels of diversity and endemism at small geographic scales - particularly in mountainous areas- underscoring the country’s role as a hotspot of Agelenidae diversity (Kaya et al. 2010, Kaya et al. 2023a, Zamani et al. 2024).

The findings of this study contribute to our understanding of *Tegenaria* and *Hellamalthonica* and highlight the need for further taxonomic and systematic investigations within the *Tegenaria/Malthonica* complex. Future research should focus on expanding the geographic and genetic sampling of these genera to assess the full extent of their diversity and evolutionary history.

## Supplementary Material

XML Treatment for Hellamalthonica kazdagensis

## Figures and Tables

**Table 1.** Leg spination of *Hellamalthonicakazdagensis* sp. nov. The letter “p” indicates paired spines.

**Legs**	**Sex**	**Fe (d-pl-rl)**	**Pa (d-pl-rl)**	**Ti (d-pl-rl-v)**	**Mt (d-pl-rl-v)**
**I**	♂	2-2-0	1-0-0	-	-
	♀	2-2-0	0-0-0	0-0-0	0-0-0-(1+1p+1p)
**II**	♂	2-1-1	1-0-0	1-2-0-2p	0-(1+1p)-1-0
	♀	-	-	-	-
**III**	♂	2-2-1	2-0-0	1-2-2-3p	(1+1p)-3-2-0
	♀	-	-	-	-
**IV**	♂	2-2-1	2-0-0	2-2-2-3p	3p-2-3-0
	♀	-	-	-	-
